# Discrimination of Explosive Residues by Standoff Sensing Using Anodic Aluminum Oxide Microcantilever Laser Absorption Spectroscopy with Kernel-Based Machine Learning

**DOI:** 10.3390/s24185867

**Published:** 2024-09-10

**Authors:** Ho-Jung Jeong, Chang-Ju Park, Kihyun Kim, Yangkyu Park

**Affiliations:** 1 Inorganic Light-Emitting Display Research Center, Korea Photonics Technology Institute (KOPTI), Gwangju 61007, Republic of Korea; hojung@kopti.re.kr; 2 Mobility Lighting Research Center, KOPTI, Gwangju 61007, Republic of Korea; pcj0027@kopti.re.kr; 3 Department of Mechatronics Engineering, Chonnam National University, 50 Daehak-ro, Yeosu 59626, Chonnam, Republic of Korea; rlarlguswkd3@naver.com; 4 Corporate Growth Support Center, Jeonnam Yeosu Industry-University Convergence Agency, 17 Samdong 3-gil, Yeosu 59631, Chonnam, Republic of Korea

**Keywords:** standoff sensing, anodic aluminum oxide cantilever, infrared spectroscopy, kernel-based machine learning, explosive residue

## Abstract

Standoff laser absorption spectroscopy (LAS) has attracted considerable interest across many applications for environmental safety. Herein, we propose an anodic aluminum oxide (AAO) microcantilever LAS combined with machine learning (ML) for sensitive and selective standoff discrimination of explosive residues. A nanoporous AAO microcantilever with a thickness of <1 μm was fabricated using a micromachining process; its spring constant (18.95 mN/m) was approximately one-third of that of a typical Si microcantilever (53.41 mN/m) with the same dimensions. The standoff infrared (IR) spectra of pentaerythritol tetranitrate, cyclotrimethylene trinitramine, and trinitrotoluene were measured using our AAO microcantilever LAS over a wide range of wavelengths, and they closely matched the spectra obtained using standard Fourier transform infrared spectroscopy. The standoff IR spectra were fed into ML models, such as kernel extreme learning machines (KELMs), support vector machines (SVMs), random forest (RF), and backpropagation neural networks (BPNNs). Among these four ML models, the kernel-based ML models (KELM and SVM) were found to be efficient learning models able to satisfy both a high prediction accuracy (KELM: 94.4%, SVM: 95.8%) and short hyperparameter optimization time (KELM: 5.9 s, SVM: 7.6 s). Thus, the AAO microcantilever LAS with kernel-based learners could emerge as an efficient sensing method for safety monitoring.

## 1. Introduction

Standoff sensing, which enables both sensing systems and personnel to be at some distance from the target substances being measured, has been a strongly sought-after capability [1] for various applications such as industrial anomaly detection [2] and greenhouse gas monitoring [3]. For example, in highly regulated areas in petrochemical plants, sensors installed for pipeline leakage detection or pump fault diagnosis should comply with stringent regulations that require extensive permissions, such as explosive-proof certification. Utilizing standoff sensing can alleviate the burden of strict regulations and ensure a safe detection of machine anomalies from outside of the hazardous zone.

Laser absorption spectroscopy (LAS) is established as one of the most sensitive technologies for quantitatively measuring hazardous chemicals [4,5]. LAS utilizes a laser as its spectroscopic light source and identifies chemical substances by detecting changes in the laser beam intensity after transmission along the optical path resulting from the laser absorption of target chemicals.

In recent years, spectroscopic techniques such as infrared (IR) and Raman spectroscopy have been intensively applied to machine learning (ML) algorithms to estimate target materials. For example, it has been used to conduct an analysis of the chemical properties of arctic soil [6], for diesel quality prediction [7], and for nasopharyngeal cancer detection [8]. Furthermore, IR spectra obtained by standoff sensing have been integrated with ML algorithms to identify plastic waste [9] and detect liquid chemicals such as diethyl phthalate and dimethyl phosphonate [10].

Rapid advances in the microfabrication process have facilitated the utilization of microcantilever LAS for standoff sensing [11,12,13]. Bimaterial microcantilevers typically demonstrate picojoule-level sensitivity [11,14], even extending to a few femtojoules [15], which allows for highly sensitive standoff sensing; IR spectra can be achieved by illuminating the microcantilever with lasers of different wavelengths, ensuring high selectivity. Also, previous research has shown that microcantilever-based standoff sensing achieved a low limit of detection, ranging from 40 ng/cm^2^ to 600 ng/cm^2^, for PETN, RDX, and TNT [13,16].

Point sensing has traditionally employed Si microcantilevers as the primary choice [17,18,19]. Nanoporous microcantilevers made of anodic aluminum oxide (AAO) are also gaining attention for their significantly larger surface area. These structures have been employed in point sensing for various applications [20,21]. Meanwhile, a nanoporous microcantilever also exhibits a much lower effective Young’s modulus [20] and thermal conductivity [22,23] than a conventional Si microcantilever owing to the presence of nanopores and the anodized oxide layer, respectively. This allows the nanoporous microcantilever to exhibit up to four times superior thermomechanical sensitivity compared to a standard Si microcantilever of the same size [20]. These advantages suggest that nanoporous microcantilevers could be applicable not only to point sensing but also to standoff sensing. However, Si microcantilevers are still predominantly employed in standoff sensing applications [9,11,13].

**Figure 1:**
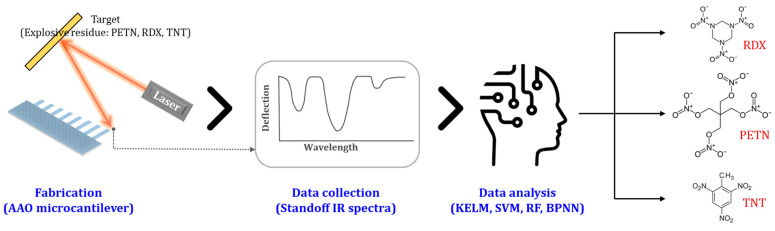
The overall scheme to show the whole idea in this work.

In this study, we develop a sensitive and selective standoff sensing system for explosive residues using AAO microcantilever LAS combined with ML. The cantilever fabrication; IR spectrum measurement of pentaerythritol tetranitrate (PETN), cyclotrimethylene trinitramine (RDX), and trinitrotoluene (TNT); and ML analysis are presented. A very thin nanoporous microcantilever with a thickness of less than 1 μm is fabricated. A wide range of standoff IR spectra are measured using a quantum cascade laser (QCL) from 5.5 to 10.5 μm. Moreover, the standoff IR spectra are analyzed using various ML models such as random forest (RF), backpropagation neural networks (BPNNs), and kernel-based ML models including support vector machines (SVMs) and kernel extreme learning machines (KELMs) to find an efficient learning model. Figure 1 depicts the overall scheme that outlines the process from the fabrication step to the data collection step and then to the data analysis.

## 2. Materials and Methods

### 2.1. Materials

Three standard explosive materials, namely, PETN, RDX, and TNT, at a concentration of 1 mg/mL, were purchased from RESTEK (Bellefonte, OH, USA) and used without further purification. 

Perchloric acid, ethanol, acetone, oxalic acid, phosphoric acid, chromium oxide (VI), nitric acid, and acetic acid were obtained from Sigma-Aldrich (Burlington, NJ, USA), and a high-purity aluminum sheet of 99.998% was purchased from Alfa Aesar (Ward Hill, MA, USA) to fabricate the AAO microcantilever. 

### 2.2. AAO Microcantilever Fabrication

**Figure 2:**
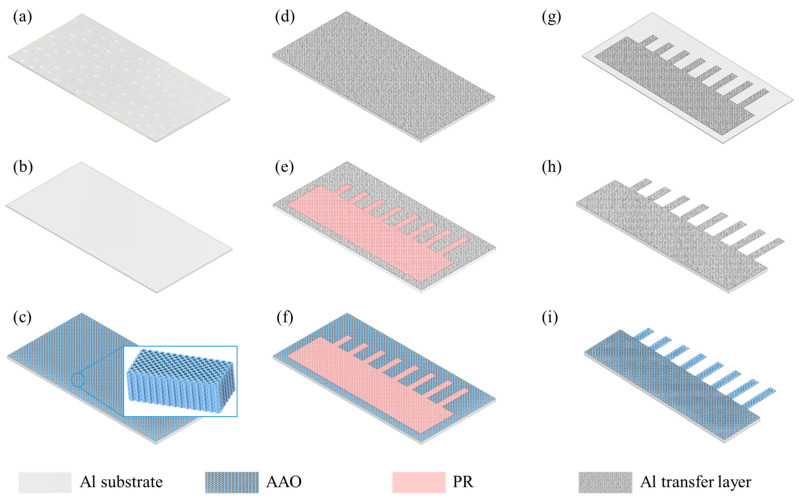
Fabrication process of AAO microcantilever. (**a**) Preparation of clean Al substrate, (**b**) electropolishing to remove contaminants on surface, (**c**) two-step anodization for nanoporous structure, (**d**) Al sputtering for transfer layer, (**e**) PR pattern by photolithography, (**f**) Al transfer layer etching, (**g**) exposed AAO etching, (**h**) electropolishing to release cantilever, and (**i**) remaining Al transfer layer etching.

AAO microcantilevers were fabricated by surface micromachining to realize cantilevers on an aluminum substrate and bulk micromachining to release the cantilevers, as shown in Figure 2. The fabrication process was referenced from previous works [12,20].

To prepare the AAO layer on the aluminum substrate, the aluminum sheet was initially cleaned using acetone, ethanol, and deionized (DI) water (Figure 2a). Subsequently, it was electropolished in a mixture of perchloric acid and ethanol solution at 5 °C by applying 20 V for 5 min to remove any contaminants from the surface and ensure a clean substrate (Figure 2b). The first anodization process was conducted at 15 °C in the oxalic acid solution for 8 h at 40 V using a DC power supply (EDU36311A, Keysight, Santa Rosa, CA, USA). This process resulted in the formation of a nonordered oxide layer. Subsequent etching with a mixture of chromium oxide (VI), phosphoric acid, and DI water at 65 °C for 6 h yielded a dimple-shaped structure on the substrate. The second anodization process was carried out under the same conditions as those in the first anodization process except for an anodization time of 10 min, which formed well-ordered nanopores on the aluminum sheet (Figure 2c). The nanopores were then expanded by immersing them in phosphoric acid solution at room temperature for 1 h, thus completing the AAO substrate fabrication. 

To fabricate the AAO microcantilevers, a 500 nm thick aluminum layer was sputtered on the AAO substrate as the transfer layer (Figure 2d), and a 1 μm thick photoresist (PR) was patterned by photolithography (Figure 2e). The deposited aluminum, unmasked by PR, was etched for 20 min using a mixture of nitric acid, acetic acid, phosphoric acid, and DI water (Figure 2f). Subsequently, the patterns of the aluminum mask were transferred to the AAO layer with 2 h of etching of the exposed AAO using phosphoric acid (Figure 2g). During AAO etching, PR was nearly removed by phosphoric acid. To release the microcantilever, the electropolishing process was conducted at 5 °C and 20 V for 1 h (Figure 2h). Next, the remaining aluminum transfer layer was clearly etched by the aluminum etchant, resulting in a suspended AAO microcantilever (Figure 2i). 

**Figure 3:**
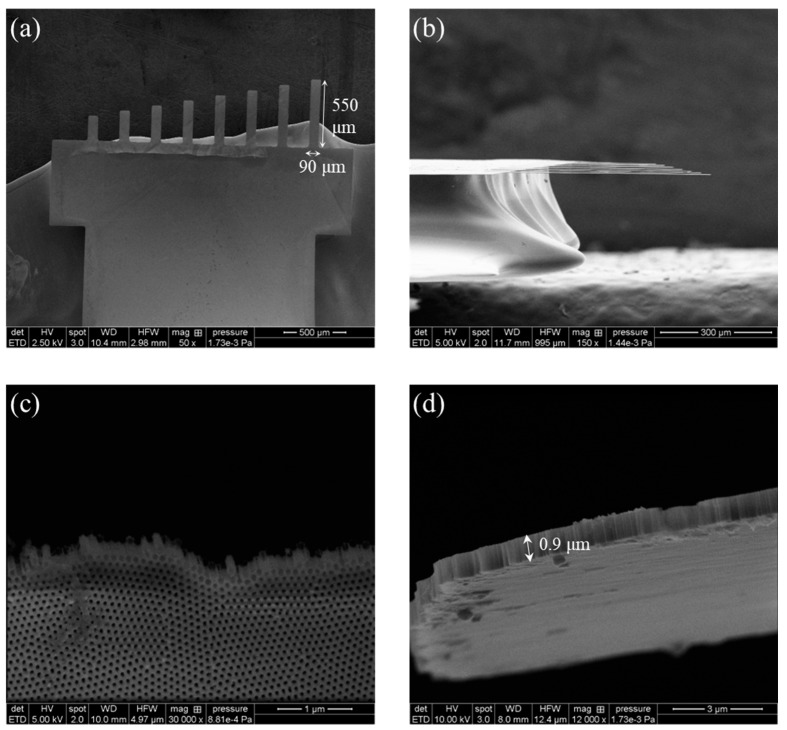
SEM images of the fabricated AAO microcantilever. (**a**) The top view, (**b**) the side view of the cantilever chip, (**c**) nanotubes forming the cantilever, and (**d**) a magnified slanted view of the cantilever. The dimensions of the longest microcantilever included a width of 90 μm, a length of 550 μm, and thickness of 0.9 μm.

Five chips including eight microcantilevers with different lengths were fabricated simultaneously from one AAO substrate. Owing to the fragile nature of the AAO microcantilevers, there would be a risk of the cantilevers breaking during the fabrication process. To ensure the availability of at least a minimal number of fabricated AAO microcantilevers, eight cantilevers of different lengths were integrated into a single chip. Additionally, to prevent significant etch rate imbalances during the electropolishing process for releasing the cantilevers, only five chips were placed on a single substrate at a time. Figure 3 shows the scanning electron microscopy (SEM; SU8010, Hitachi, Japan) images of the AAO microcantilevers in one of the chips from different viewing angles and magnifications. Figure 3a,b display eight AAO microcantilevers with a width of 90 μm and lengths ranging from 200 to 550 μm. Figure 3c illustrates the nanotubes constituting the microcantilever. The pore diameter and pore pitch were approximately 55 and 100 nm, respectively; the porosity was calculated to be 0.274. An extremely thin thickness of 0.9 μm can be confirmed in Figure 3d.

A metal layer under the AAO microcantilever is required for a bimetallic effect, where two materials with different thermal expansion coefficients cause the cantilever to bend when exposed to heat. Additionally, this metal layer is necessary for the optical lever technique, which uses a red laser to measure the deflection of the cantilever. However, once the thickness of the metal layer exceeds a certain threshold, the deformation of the cantilever decreases because of the increase in spring stiffness and the reduction in thermal resistance. To maximize the thermomechanical effect, Au was sequentially sputtered in increments of 20 nm (20, 40, 60, and 80 nm) under one of the five AAO cantilever chips while measuring the baseline IR spectrum at each Au thickness. The largest signal was observed at a Au thickness of 60 nm. Consequently, the remaining four chips were sputtered with a 60 nm thickness of Au, and the longest AAO microcantilever (90 × 550 × 0.9 μm) was utilized for the standoff sensing of the explosive residues. The optimized Au thickness will be further discussed in Section 3.1.

### 2.3. The Resonant Frequency Measurement of the AAO Microcantilever Comprising AAO Nanopores without a Au Layer

A laser Doppler vibrometer (LDV; MSV300, Polytec, Germany) was used to measure the resonant frequency of the transparent AAO microcantilever comprising AAO nanopores without a Au coating. The resonant frequency was utilized to estimate the effective Young’s modulus and spring stiffness of the AAO microcantilever, which is discussed in Section 3.1.

### 2.4. Standoff LAS Setup

**Figure 4:**
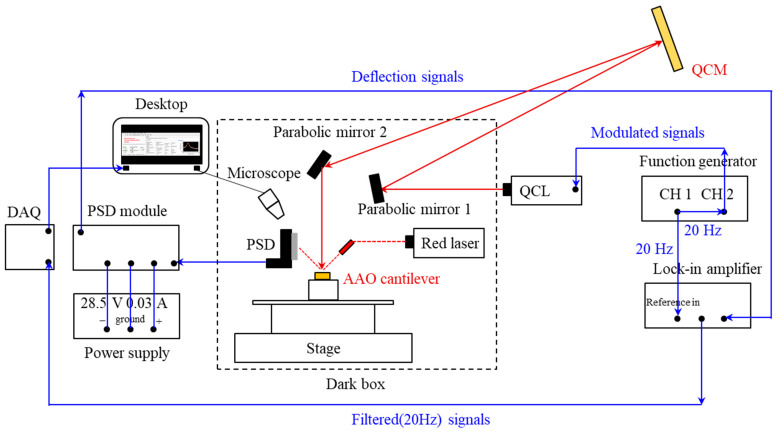
The experimental setup for the standoff LAS using the AAO microcantilever. The optical path length from the QCM to parabolic mirror 2 is 20 cm, and that to the AAO cantilever is 30 cm.

Figure 4 shows the experimental setup for the standoff sensing of the explosive residues. Three types of explosive solutions of 50 μL were dispensed onto individual quartz crystal microbalances (QCMs; Inficon, Bad Ragaz, Switzerland) and evaporated for a day. Ten QCM samples were prepared for each explosive material, totaling 30 QCM samples. The deposited surface concentrations (Δ*m*) of the explosive residues were estimated from the change in the resonant frequency (Δ*f*) of the QCM according to the Sauerbrey equation [24]:

(1) $$∆f=-\frac{2{f_{0}}^{2}}{A\sqrt{\rho_{q}\mu_{q}}}∆m,$$

where *f_0_*, *A*, *ρ_q_*, and *µ_q_* are the initial resonant frequency, piezoelectrically active crystal area (1 cm^2^), density of quartz (2.648 g/cm^3^), and shear modulus of quartz for AT-cut crystal (2.947 × 10^11^ g/cm·s^2^), respectively. The QCM was fixed in a crystal holder, and the resonant frequency shift was measured using a QCM instrument (QCM200, Standford Research Systems, Sunnyvale, CA, USA). 

Twenty-hertz pulses were generated on channel 1 of a two-channel function generator (33500, Keysight, Santa Rosa, CA, USA), and these 20 Hz pulses were used to generate 20 Hz-modulated 200 kHz pulses (5% duty cycle) on channel 2. Subsequently, the quantum cascade laser (QCL; LaserTuneTM, Block Engineering, Marlborough, MA, USA) driven by the modulation signals emitted the pulsed IR laser toward the target explosive residues on the QCM through parabolic mirror 1. The reflected IR laser from the QCM was directed toward the Au-coated AAO microcantilever through parabolic mirror 2. The AAO microcantilever was sequentially exposed to an IR wavelength band ranging from 5.5 μm to 10.5 μm with 171 measurement points in a single spectrum. The optical path length between the QCM and parabolic mirror 2 was 20 cm, and the distance between parabolic mirror 2 and the AAO microcantilever was 10 cm. 

An optical lever method was used to measure the deflection of the AAO microcantilever. A red laser (LDM635, Thorlabs, NJ, USA) was made incident on the Au-coated AAO microcantilever and redirected to a position-sensitive detector (PSD; On-Trak, Irvine, CA, USA). The deflection signals of the AAO microcantilever from the PSD were filtered using a lock-in amplifier (SR850, Stanford Research Systems, Sunnyvale, CA, USA) to exclude all frequency components except the reference frequency of 20 Hz. Then, the filtered signals were transferred to a data acquisition board (DAQ; National Instruments, Austin, TX, USA). A customized LabVIEW (National Instruments, Austin, TX, USA) program was employed to collect the data and produce standoff IR spectra on a desktop computer.

### 2.5. ML Models

The standoff IR spectra of the explosive residues were converted into a labeled dataset, which was utilized to train and evaluate several ML models. Four ML models chosen for the discriminating explosive residues were the KELM, SVM, RF, and BPNN, all recognized for their robust performance in classification tasks. Meanwhile, established references [25,26] provide comprehensive coverage of traditional ML approaches such as SVMs, RF, and BPNNs. Thus, in this section, we explore the principles of KELM, a relatively recent ML model, providing an overview of its core concepts.

Extreme learning machine (ELM), proposed by Huang et al. [27], is a simple and efficient learning algorithm using random feature mapping and calculating analytical solution. Suppose a single-layer feedforward neural network (SLFN) is trained with *L* hidden neurons and an activation function (*g*) to learn *n* distinct samples (**x**_i_, **t**_i_), where **x**_i_ = [*x_i1_*, *x_i2_*,..., *x_id_*]^T^ ∈ **R**^d^ is a d-dimensional input vector, **t**_i_ = [*t_i1_*, *t_i2_*,..., *t_ic_*]^T^ ∈ **R**^c^ is a one-hot vector of the target class, and *c* is the number of classes. Then, given the input vector **x**, the output of SLFNs with L hidden nodes can be written as

(2) $$f_{SLFN}\left(x\right)=\sum_{j=1}^{L}\beta_{j}h_{j}(w_{j},b_{j},x)=h(x)\beta ,$$

where h_j_ = g(**w**_j_ · **x** + *b_j_*) denotes the output of the *j*^th^ hidden neuron and $h\left(x\right)=$ [$h_{1}(w_{1},b_{1},x$), $h_{2}(w_{2},b_{2},x$),..., $h_{L}(w_{L},b_{L},x)$] is a hidden layer vector; **β**_j_ = [*β_j_*_1_, *β_j_*_2_,..., *β_jc_*]^T^(*j =* 1, 2,..., *L*) denotes the output weight vector connecting the *j*^th^ hidden neuron and output neurons and **β** = [**β**_1_, **β**_2_,..., **β**_L_]^T^ is the output weight matrix; **w**_j_ = [*w_j_*_1_, *w_j_*_2_,..., *w_jd_*]^T^ is the input weight vector connecting the *j*^th^ hidden neuron and input neurons; and *b_j_* denotes the bias of the *j*^th^ hidden neuron. Assuming that the outputs can be approximated with zero errors given n samples, the output target (**T**) in matrix form can be expressed as [28]

**T** = **Hβ**, (3)

where **H** = [**h**(**x_1_**), **h**(**x_2_**),..., **h**(**x_n_**)] is a hidden layer matrix; **T** = [**t**_1_, **t**_2_,..., **t**_n_]^T^ is the output target matrix. The input weights and biases in the ELM model are randomly selected. Then, the process of determining the output weight matrix is as simple as finding the minimum norm least-squares solution as follows:

(4) $$\underset{\beta }{\min}\left\|H\beta -T\right\|,$$

and the optimal estimation of the output weight matrix can be expressed as Equation (5):

(5) $$\beta =H^{\text{†}}T,\;H^{\text{†}}=\;H^{T}{\left(\frac{I}{C}+\;H^{T}H\right)}^{-1},$$

where **H^†^** denotes the Moore–Penrose generalized inverse of matrix **H**, **I** is an identity matrix, and C represents the regularization parameter. Thus, the ELM model f_ELM_ can be represented by

(6) $$f_{ELM}\left(x\right)=h(x)H^{T}{\left(\frac{I}{C}+H^{T}H\right)}^{-1}T.$$

In contrast to traditional ML models, such as BPNNs, the input weights and biases in ELM are randomly chosen, and the corresponding output weights are analytically computed instead of obtained through iterative optimization methods such as backpropagation. Consequently, the ELM can save most of the learning time traditionally spent in tuning the parameters, contributing to efficient computation. However, owing to the random selection of input weights and biases in ELM, the forecasting results are not the same even under the same parameter setting, which results in unstable forecasting performance [29]. 

**Figure 5:**
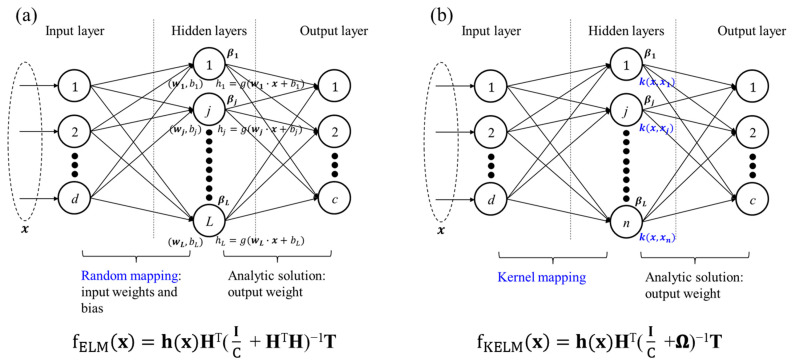
A comparison between the ELM and KELM networks. (**a**) ELM and (**b**) KELM. *d*, *L*, *c*, and *n* in the last neuron of each layer represent layer dimensions.

For the robust prediction accuracy of ELM, KELM was proposed by Huang et al. [30]. KELM incorporated a kernel function (k) into ELM, replacing random feature mapping with kernel mapping to effectively address the unstable prediction performance. The kernel matrix (**Ω**) can be defined according to Mercer’s theorem [31]:

(7) $$\Omega =H^{T}H,$$

where Ω_p,q_ = k(**x**_p_, **x**_q_), *p*, q = 1 to n. Then, by replacing $H^{T}H$ in Equation (6) with Ω from (7), the KELM model f_KELM_ can be obtained:

(8) $$f_{KELM}\left(x\right)=h(x)H^{T}{\left(\frac{I}{C}\;+\;\Omega \right)}^{-1}T,$$

where $h(x)H^{T}$ = [k(**x**, **x**_1_), k(**x**, **x**_2_),..., k(**x**, **x**_n_)]^T^. Figure 5 depicts the network structures of ELM and KELM for a comparison.

## 3. Results and Discussion

### 3.1. Mechanical Property Estimation of AAO Microcantilever Consisting of AAO Nanopores 

**Figure 6:**
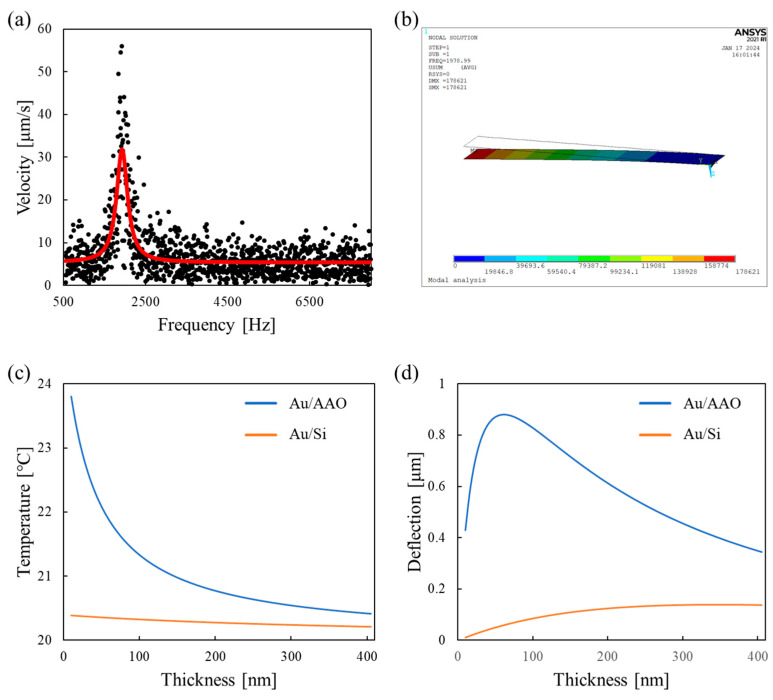
A mechanical property estimation of the AAO microcantilever. (**a**) A scatter plot measured using the LDV. The red line represents the Lorentzian fitted curve. (**b**) The modal analysis results from the FEA. The mode shape at the first resonant frequency of 1978.99 Hz is depicted. The thermal–structural coupled analysis results. (**c**) The maximum temperature comparisons and (**d**) the maximum displacement comparison between the Au/AAO and Au/Si microcantilevers.

Prior to depositing the Au layer on the AAO microcantilever, the resonant frequency was measured to estimate the effective Young’s modulus of the AAO microcantilever comprising pure AAO nanopores. Figure 6a shows the frequency response of the highly transparent AAO microcantilever, as measured by an LDV, along with its Lorentzian fit curve. The fitting process involved minimizing the sum of the squared residuals between the experimental data points and the Lorentzian model. By adjusting the parameters of the Lorentzian function, such as the spectral linewidth, the best-fit curve was obtained, as illustrated in Figure 6a. The resonant frequency was determined to be 1931 Hz by Lorentzian fitting to the measured velocity. The relationship between the resonant frequency and the effective Young’s modulus can be expressed as Equation (9) [21]:

(9) $$f_{n}={\beta_{n}}^{2}\frac{1}{2\pi \sqrt{12}}\frac{t}{l^{2}}\sqrt{\frac{E}{\rho }}$$

where *f_n_* denotes the *n*^th^ mode resonant frequency; *β_n_* is the *n*^th^ mode eigenvalue (*β_1_* = 1.875); *t* and *l* are the thickness and length of the cantilever, respectively; and *E* and *ρ* are the effective Young’s modulus and effective density, respectively. The effective density of the AAO microcantilever was calculated by multiplying the aluminum oxide density and porosity of the nanoporous microcantilever. Accordingly, the effective Young’s modulus of our AAO microcantilever was determined as 47.2 GPa. The Young’s modulus of the AAO microcantilever was much lower than that of traditional Si microcantilevers (130 GPa). Meanwhile, regarding the resonant frequency estimation, the approach utilizing a 3D simulation would be helpful [32].

A finite element analysis (FEA; ANSYS APDL, ANSYS, Canonsburg, PA, USA) was performed to verify the estimated effective Young’s modulus and the effective density. A modal analysis was performed using shell elements to effectively model the thin thickness of the cantilever. The effective Young’s modulus experimentally determined using the LDV and the effective density estimated from the porosity were utilized as the material properties of the AAO microcantilever in the FEA. As shown in Figure 6b, a resonant frequency of the AAO microcantilever obtained from the FEA was 1978.99 Hz, which closely matched the experimental results with an approximately 2% error. A modal analysis of a Si microcantilever was also conducted to numerically compare the spring constant of the AAO microcantilever with that of the corresponding Si microcantilever with identical dimensions. The spring constants were extracted on the basis of the mass and first mode resonant frequencies that were obtained from the simulation results. The spring constant of the AAO microcantilever (18.95 mN/m) was significantly reduced approximately three times compared to that of the corresponding Si microcantilever (53.41 mN/m). 

In addition, a thermal-structural coupled analysis was performed to evaluate the thermomechanical sensitivity of the Au/AAO and Au/Si bimetallic microcantilevers. The applied power increased the temperature of the bimetallic microcantilever, which has different thermal conductivity and expansion coefficients, leading to cantilever deformation. Figure 6c,d represent the maximum temperature and z-directional displacement of the cantilevers, respectively, as a function of Au’s thickness in 5 nm increments. Because the thermal resistance of AAO is much higher than that of Si [23,33], the Au/AAO cantilever showed higher temperatures than the Au/Si cantilever. Moreover, the Au/AAO bimetallic cantilever, with a higher temperature, a lower spring constant, and a larger difference in thermal expansion coefficients, exhibited superior thermomechanical sensitivity compared to the Au/Si cantilever. The maximum thermomechanical sensitivity was approximately 500 nm/K at a Au thickness of 60 nm. This optimal Au thickness corresponded to the experimentally determined thickness explained in Section 2.2.

### 3.2. Standoff IR Spectra Measurements of Explosive Residues

The standoff IR spectra were measured with the bimetallic AAO microcantilever using the experimental setup illustrated in Figure 4. After measuring the reference IR spectrum with a clean QCM, the IR spectrum of the explosive residue was acquired. Then, the differential IR spectrum was obtained by subtracting the IR response of the AAO microcantilever with a target explosive on a QCM from the IR response measured with the clean QCM alone. For each type of explosive material, 10 samples were prepared to be positioned on individual QCMs. The average surface concentrations of PETN, RDX, and TNT were estimated to be 13.7, 7.9, and 8.6 μg/cm^2^, respectively, by QCM measurements. The IR spectra for each sample were obtained at 12 different positions. Thus, the total number of the differential IR spectra for 30 samples of the explosive residues was 360. 

**Figure 7:**
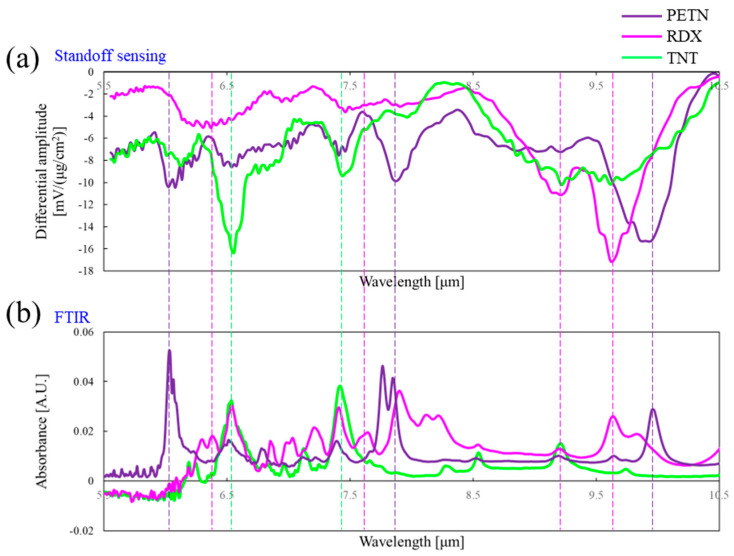
A comparison between the differential IR spectra measured using our AAO microcantilever LAS and the standard FTIR spectra for PETN, RDX, and TNT. (**a**) The differential IR spectra normalized by the surface concentrations and (**b**) the FTIR spectra.

Figure 7a displays the average differential IR spectra for PETN, RDX, and TNT normalized by corresponding surface concentrations. The standoff IR spectra obtained from our AAO microcantilever LAS were clearly discriminated for each explosive residue, indicating the high selectivity of our sensor. The presence of explosive molecules on the QCM surface led to the absorption of IR energy at specific standoff IR wavelengths. Consequently, the redirected IR power to the AAO microcantilever was diminished, resulting in negative differential IR spectra.

Standard Fourier transform infrared (FTIR) spectra were also measured for PETN, RDX, and TNT on the QCMs using the attenuated total internal reflection mode of an FTIR spectrophotometer (IRAffinity, SHIMADZU, Kyoto, Japan), as depicted in Figure 7b. The majority of characteristic peaks between the standoff IR spectra measured using our sensor and the standard FTIR closely aligned with each other. The peaks at 6.06, 7.81, and 9.94 μm corresponded to PETN, while those at 6.34, 7.6, 9.17, and 9.61 μm were from RDX. Additionally, the peaks at 6.55 and 7.46 μm could be identified as TNT signatures. According to previous reports [34,35,36,37], the peaks at 6.06, 6.34, and 6.55 μm were attributed to NO_2_ asymmetric stretching in PETN, RDX, and TNT, respectively. The peaks at 7.46 and 7.81 μm were associated with NO_2_ symmetric stretching in TNT and PETN, respectively, while the peak at 7.6 μm in RDX was indicative of CH_2_ bending. In addition, the peaks at 9.17 and 9.61 μm in RDX were ascribed to CH_2_ twist, and the peak at 9.94 μm in PETN was attributed to CO stretching. This comparison with the standard FTIR not only provided experimental validation for the standoff spectra but also demonstrated the high selectivity of our sensor.

Considering that microcantilever-based standoff sensing techniques have been applied to various applications, including explosive residue detection—not only for the three substances used in this experiment but also for other residues like dimethyl methyl phosphonate [16]—and for polymer discrimination among materials such as polydimethylsiloxane, polymethyl methacrylate, polyvinyl alcohol, and SU-8 photoresist [11], our proposed method could be even more broadly applicable.

### 3.3. ML Analysis

A set of 120 normalized differential IR spectra for each type of explosive residue was assembled to form a total of 360 labeled IR spectra spanning 5.5–10.5 μm. Consequently, a dataset comprising 360 instances and 171 features was used to feed the KELM, SVM, RF, and BPNN. The numbers of randomly allocated instances for model training and validation were 216 and 72, respectively, while the remaining 72 instances were designated for the testing dataset to assess model performance (training/validation/testing = 80:20:20). All standoff differential IR spectra were normalized by z-score preprocessing. A principle component analysis (PCA) was carried out to increase the computational efficiency. The PCA extracted the first three principal components that accounted for 72.3% of the total variance.

The hyperparameters of all ML models except the BPNN were optimized using a grid search on the training and validation datasets. Two hyperparameters with 50 variables each were set for KELM, SVM, and RF. This enabled 250 iterations to be performed for the training and validation process equally throughout these three ML models, facilitating a comparison of their computational efficiency. The hyperparameters chosen for KELM and SVM were the kernel parameter and the regularization parameter. A radial basis function was employed as their kernel function. Furthermore, the number of estimators and the maximum depth were selected as the hyperparameters for RF.

Compared to the KELM, SVM, and RF, the BPNN requires the specification of more hyperparameters to compile the neural network model. For example, besides specifying the number of neurons and hidden layers for the network structure, other hyperparameters such as the epoch and batch size need to be specified. Consequently, optimizing numerous hyperparameters via the grid search could lead to substantial computational costs. Bayesian optimization (BO) offers an alternative approach, enabling hyperparameter tuning in fewer iterations by constructing a probabilistic surrogate model based on Gaussian processes [38,39]. This method is particularly suitable for models with a large number of hyperparameters. Thus, BO was utilized to determine the hyperparameters for the BPNN. The Adam optimizer was employed, with the ReLU activation function in the hidden layers and the Softmax activation function in the output layer, while the number of hidden layers, number of neurons in each hidden layer, epoch, batch size, and learning rate were tuned via the BO process. 

**Figure 8:**
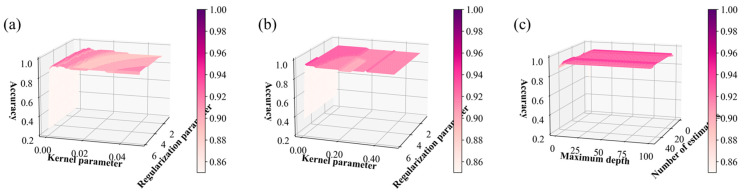
Validation accuracy with respect to the hyperparameters for the (**a**) KELM, (**b**) SVM, and (**c**) RF. The vertical axis denotes the validation accuracy, and the horizontal axis depicts the hyperparameters. The color bars represent the validation accuracy. The optimization conditions including the optimal hyperparameters are summarized in Table 1.

**Figure 9:**
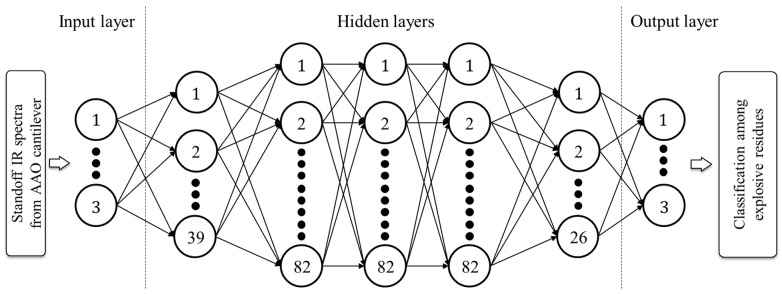
BPNN structure determined using BO. The number in the final neuron at each layer indicates the total number of neurons within that layer. The number of neurons and layers for the optimal validation accuracy can be seen.

Figure 8 depicts the validation accuracy with respect to the different hyperparameter pairs for the KELM, SVM, and RF throughout the iterative grid search process. By examining the surface gradient of the validation accuracy plot in Figure 8a,b, it can be confirmed that the kernel parameter was more decisive as a hyperparameter compared to the regularization parameter in KELM and SVM. RF maintained a relatively consistent validation accuracy regardless of changes in the hyperparameters, as shown in Figure 8c. The optimal hyperparameters for KELM were a kernel parameter of 0.006 and a regularization parameter of 1, while for SVM, the kernel and regularization parameters were 0.01 and 4.2, respectively. The number of estimators and the maximum depth were determined as 17 and 5, respectively, for RF. Figure 9 illustrates the optimized BPNN structure obtained using the BO process. Further, the optimal epoch, batch size, and learning rate were determined as 327, 22, and 0.0004, respectively. The optimization processes for the hyperparameters and their results are summarized in Table 1.

**Figure 10:**
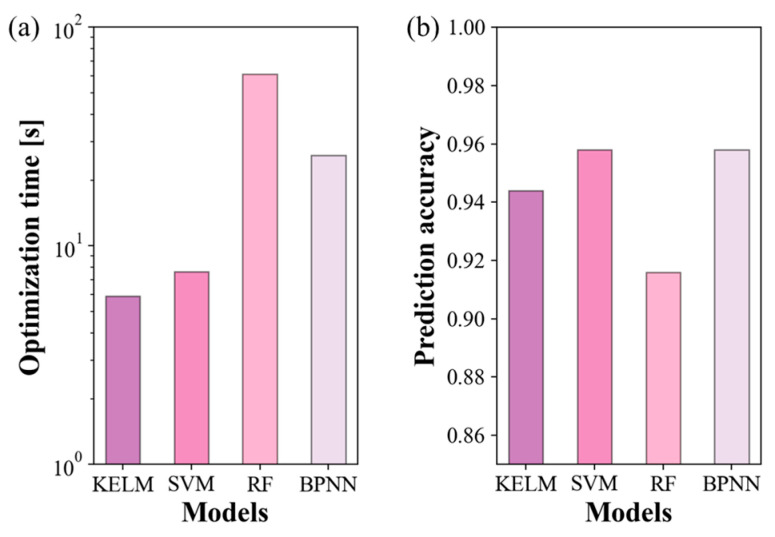
Performances of ML models: KELM, SVM, RF, and BPNN. (**a**) Optimization time and (**b**) prediction accuracy. KELM showed highest optimization speed, and SVM and BPNN demonstrated highest prediction accuracy.

All ML models showed high validation accuracies at the optimal hyperparameters: 93.1% for KELM, 94.4% for SVM and RF, and 91.7% for BPNN. However, there were significant differences in the optimization times among the ML models, as depicted in Figure 10a. Though BPNN involves a larger number of hyperparameters compared to RF, the optimization time for BPNN using BO was shorter than that for RF using grid search. The relatively rapid determination of the optimal values of BPNN was attributed to BO. However, it is noteworthy that the kernel-based ML models such as the KELM and SVM using the grid search demonstrated much shorter optimization times than the BPNN despite using BO. The optimization speed of the KELM was the highest among all the ML models. Specifically, the optimization time for the KELM (5.9 s) was 10 times shorter than that of RF (61.2 s) and 4.4 times shorter than that of the BPNN (26 s), respectively. The longer optimization time for RF and the BPNN would be due to the increased complexity in model training. RF involves multiple decision trees, while a BPNN requires iterative backpropagation across multiple layers and neurons, both of which are computationally intensive.

The prediction accuracy was assessed using the testing dataset, as shown in Figure 10b. Consistent with the validation accuracy, all ML models exhibited high prediction accuracies exceeding 90%, implying that our AAO microcantilever LAS provided a highly differentiable dataset. The SVM and BPNN yielded the highest prediction accuracy of 95.8%, followed closely by KELM with an accuracy of 94.4%. RF exhibited an accuracy of 91.7%, which was slightly lower than those of the other ML models.

**Figure 11:**
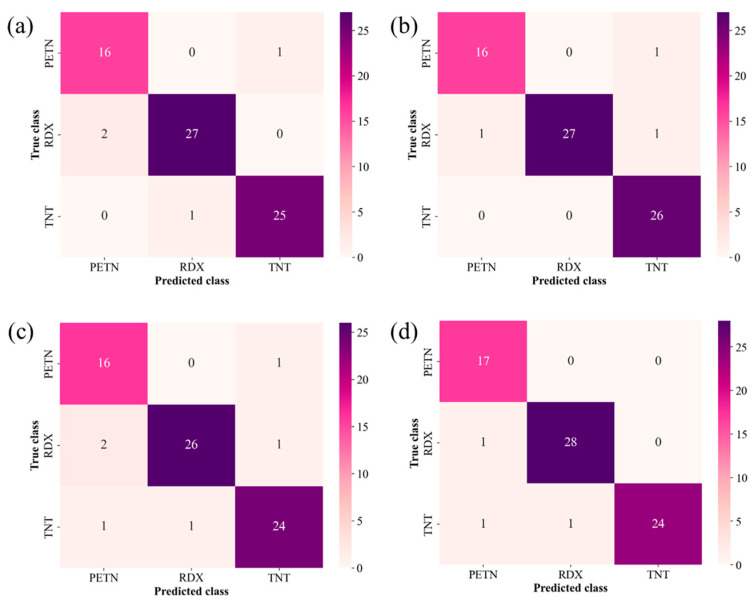
Confusion matrices for (**a**) KELM, (**b**) SVM, (**c**) RF, and (**d**) BPNN. Performance metric scores were derived from matrices; their scores are summarized in Table 2.

In addition to the optimization speed and prediction accuracy, confusion matrices were acquired for a further performance analysis of the classification models. The macro sensitivity, specificity, precision, and F1 score were evaluated using the confusion matrices, as depicted in Figure 11. The performance metric scores calculated for all the models are summarized in Table 2. While all models demonstrated robust performance, the SVM and BPNN consistently achieved good scores in the performance metrics. Specifically, the SVM excelled in precision, whereas the BPNN performed well in sensitivity. In the other metrics, the performances of the SVM and BPNN were comparable. Although the KELM and RF showed relatively lower performance, they still demonstrated high performance with over 90% in all metrics. Notably, the macro specificity exceeded 95% for all models. This means that the combination of our AAO microcantilever LAS and ML models exhibited few false-positive errors. Thus, this result suggests that our sensor is reliable, particularly under false alarms that incorrectly indicate the presence of explosive residues despite their absence.

The performance metric scores were generally high across all ML models, while the SVM and KELM were found to be superior to RF and the BPNN in terms of the hyperparameter optimization time. Both the SVM and KELM employ a kernel function, enabling the transformation of input data into a higher-dimensional space without explicit feature space expansion. Thus, nonlinear relationships of the dataset were effectively handled to increase generalization capability so that both high metric scores and a high optimization speed were achieved. This outcome indicates that SVM and KELM can offer a balance between accuracy and simplicity, enabling quick learning while delivering competitive accuracy. This enables our sensor to be equipped with not only high sensitivity and selectivity but also efficient learning. Furthermore, the versatility of our sensor makes it suitable for a wide range of applications, including embedded systems, where computational resources might be limited.

## 4. Conclusions

In this study, we developed an AAO microcantilever LAS combined with ML to differentiate among the explosive residues of PETN, RDX, and TNT. Details regarding fabrication, experiment, ML analysis for an AAO microcantilever LAS were presented. The spring constant of the AAO microcantilever was three times smaller than that of the general Si microcantilever, and the FTIR spectra of each target material agreed well with the standoff IR spectra measured using our sensor, which validates the high sensitivity and selectivity of our sensor. Among the four ML models, the KELM and SVM, which are based on a kernel function, exhibited high classification performance in terms of both high prediction accuracy and high optimization speed. The SVM achieved a prediction accuracy of 95.8% in classifying the explosive residues with a hyperparameter optimization time of 7.6 s, and the KELM reached a prediction accuracy of 94.4% with a hyperparameter optimization time of 5.9 s.

## Figures and Tables

**Table 1:** The optimization processes for the hyperparameters and their results.

	KELM	SVM	RF	BPNN *
Optimization method	grid search	grid search	grid search	BO
Optimal hyperparameters	(regularization, kernel) = (0, 0.006)	(regularization, kernel) = (4.2, 0.01)	(depth, estimators) = (5, 17)	(epoch, batch, learning rate) = (327, 22, 0.0004)
Optimization time	5.9 s	7.6 s	61.2 s	26 s
Validation accuracy	93.1%	94.4%	94.4%	91.7%

* The optimal hyperparameters for the number of layers and neurons are illustrated in Figure 9.

**Table 2:** Performance metrics for classification models.

	KELM	SVM	RF	BPNN
Macro sensitivity	94.5%	95.7%	92.0%	96.3%
Macro specificity	97.3%	97.9%	96.0%	98.0%
Macro precision	93.8%	95.7%	90.9%	95.3%
Macro F1 score	94.1%	95.6%	91.4%	95.7%

## Data Availability

Data are contained within the article.
